# The Birth-Death-Mutation Process: A New Paradigm for Fat Tailed Distributions

**DOI:** 10.1371/journal.pone.0026480

**Published:** 2011-11-01

**Authors:** Yosef E. Maruvka, David A. Kessler, Nadav M. Shnerb

**Affiliations:** Department of Physics, Bar Ilan University, Ramat-Gan, Israel; Tel Aviv University, Israel

## Abstract

Fat tailed statistics and power-laws are ubiquitous in many complex systems. Usually the appearance of of a few anomalously successful individuals (bio-species, investors, websites) is interpreted as reflecting some inherent “quality” (fitness, talent, giftedness) as in Darwin's theory of natural selection. Here we adopt the opposite, “neutral”, outlook, suggesting that the main factor explaining success is merely luck. The statistics emerging from the neutral birth-death-mutation (BDM) process is shown to fit marvelously many empirical distributions. While previous neutral theories have focused on the power-law tail, our theory economically and accurately explains the entire distribution. We thus suggest the BDM distribution as a standard neutral model: effects of fitness and selection are to be identified by substantial deviations from it.

## Introduction

Survival of the fittest or of the luckiest? The answer depends on the subject considered. Out of ten pairs of pants bought a year ago, the survivors are perhaps those made of a better material; if wineglasses are considered, persistence is mainly a matter of luck. In the absence of prior knowledge, statistics must be used in order to identify the role of fortune: wineglass life expectancy, for example, is described by an exponential distribution. Strong deviations from this statistics indicate to what extent “death” is a result of accumulated wear, rather than from uncorrelated random events.

In many complex systems, though, it is hard to identify relative role of fortune. Large differences in success (of investors or authors) or abundance (of bio-species) do not necessarily reflect the “quality” or the “fitness” of the rich and the frequent. Huge abundance fluctuations may be a result of accumulation of stochastic events, as exemplified by the uneven statistics of surnames in society [1].

The schism between the “neutral” (stochastic) and the “fitness” (deterministic) outlooks is most strongly pronounced in the theory of evolutionary dynamics [2]. Darwin condemned those who “attribute … (species') proportional numbers to what we call chance. But how false a view is this! [3]” and held that the main factor shaping eco-communities is natural selection. The opposite view, that random drift plays the major role in evolution — both on the molecular (Kimura's neutral evolution [4]) and the ecological (Hubbell's community drift model [5]) levels — has sparked a series of ongoing hot and emotional debates.

In economy and social sciences the deterministic approaches tend to emphasize the tremendous inequality in income and wealth, say, as reflecting underlying “quality” (from prudence to crookedness) differences. The opposing neutral approach [6] have recently found a prominent outspoken, Nassim Taleb. In his books [7], [8] he maintains that the weight of unpredictable events (what he calls “black swans”) is overwhelming in determining economic and social success.

Purely deterministic and purely stochastic theories are both oversimplifications. The real scientific problem is to find the relative weight of chance versus fitness. The assumption of neutral dynamics is most useful as a null hypothesis, with which empirical statistics should be compared. Nowadays this role is played by the Yule-Simon statistics [9]–[11], or its approximation by a simple power law [12], [13]. In the following we briefly review Yule's model and point out its major shortcoming. We suggest a correction that yields different statistics and show that the new distribution fits many “canonical” empirical datasets very nicely.

**Figure 1:**
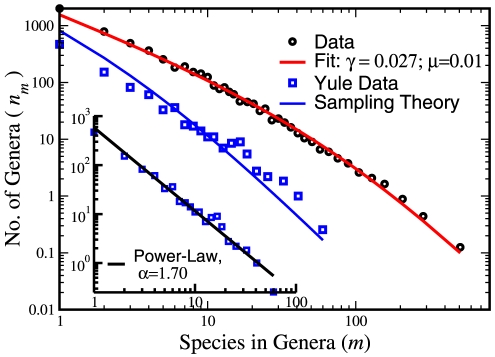
Species within genera statistics for Cerambycinea beatles. The original species within genera statistics used by Yule (blue squares), based on 1024 genera known at 1925 for the Cerambycinea family (down left). On a log-log scale this graph looks very much straight, suggesting a power-law statistics (black line). In the main figure, the black circles show the contemporary statistics as obtained for 4411 genera (27221 species of Cerambycinea [24]), where a pronounced “shoulder” appears. The red line is the best fit of BDM function (2), 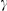 is the diversification rate and 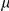 reflects the chance of a new species to initiate a new genus. The blue line shows the prediction of our theory for a sample of 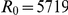 species chosen at random out of the 22271 known today with the same 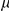 and 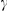, as obtained from Eq. 3. This is now a prediction *without any fitting parameters*, to be compared with the original Yule statistics.

Yule-Simon theory [9] arose from a study of the the highly skewed distribution of biological species within genera. One of the graphs studied by Yule — for the family of long-horn beatles Cerambycinea — is plotted in the left inset of Fig. 1. This is a Pareto plot showing 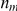, the fraction of genera with 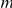 species, vs. 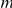 on a log-log scale. One observes a few “wealthy” genera to which many species belong, and many “poor” genera with apparent linear dependence that suggests a power-law distribution.

Yule's neutral model posited that the rate of speciation is fixed for all species. Upon speciation, the new species stays in the same genus with probability 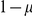. 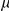, the chance that the offspring species branches out to form a new genus, is also fixed, ensuring perfect neutrality (no fitness). This simple process generates a steady state distribution that converges rapidly to a power law for the relative species abundance 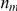,
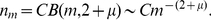
(1)where 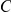 is a normalization factor. Note that this fat-tailed distribution has nothing to do with the “quality” differences among species, instead it is a result of the multiplicative character of the noise.

As pointed out by Herbert Simon [10], Yule's argument goes far beyond its original context. Simon considered power-laws for the number of occurrences of words in a text, scientific publications and wealth distribution. Subsequently, the appearance of power-laws has been recognized as a fundamental feature of eco-, econo-, bio- and socio-systems, with countless of examples from protein family statistics [14], surname abundance ratio [1], [15], internet connections [16], firm sizes [17], casualties in terror attacks [18] and so on. In addition the common scenario considered in the new popular theory of scale free networks - the preferential attachment dynamics - is indeed mathematically equivalent to Yule's process [see methods (A)] where small families are generated by a source, not by mutations [11].

## Results and Discussion

As a starting point for the presentation of our new neutral model, let us stick for the moment to the original context of Yule theory, the species within genera statistics. The main panel of Figure 1 reveals a major failure of the Yule-Simon model. The original distribution observed by Yule for Cerambycinea beatles, based on the 1024 genera (5719 species) is compared with the current data with 27221 species and 4411 genera. Clearly, something bad has happened to the simple power-law: it characterizes now only the tail of the distribution, and a very pronounced “shoulder” appears for the small genera.

This shoulder appears in almost any fat-tailed distribution [11]. Accordingly, a “power law fit” indeed involves *two parameters*: a threshold 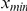 marking the end of the shoulder and the tail's slope. Unfortunately, the large argument tail tends to be of poor quality, noisy, brutish and short. Very rarely one finds a reliable dataset that allows for a good quality fit. Indeed, a recent metaanalysis by Clauset, Shalizi and Newman [19] reveals that, among 20 canonical datasets considered, only in one case a power law fit is really convincing and in most cases other distributions are doing better.

We suggest that these obstacles reflect an essential shortcoming of the Yule-Simon theory: the neglect of “death” events. In reality species go extinct, individuals die and links break down, yet in the Yule-Simon theory this never happens. A death process cannot be taken into account by simply introducing a net birth rate; it also accounts for the stochastic extinction of existing families (genera). Yule theory thus overestimates the fraction of small families, which explains the typical “shoulder” that appears at small 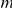
's.

Recently Manrubia and Zannete [1] studied the distribution of surnames in a population, using a model which is a specific example of the birth-death-mutation (BDM) process (see also [20]). We [15] then extended these results, showing that the resulting distribution is independent of the particular details of the process. In the spirit of Simon's realization that the Yule model results are applicable in a much broader context, we here propose, and demonstrate by numerous examples, that the BDM process and its resulting statistics should be applicable to a very wide range of empirical datasets.

### BDM statistics: results and applications

Here is a list of the main results for the statistics of the BDM process, where the total population is growing/decaying at rate 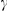. In the supplementary material we resent a detailed description of the BDM dynamics and establish the equivalence between this process and preferential attachment [16] with the possibility of link removal.

1. The probability distribution function (the chance 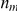 to pick at random a family of size 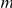) is described by the Kummer function 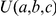 [21].
  - If the growth rate 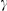 is larger than the mutation rate 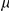, an asymptotic power-law tail appears:
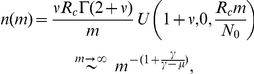
where 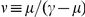 and 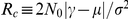, 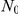 is the current population size.
  - For 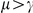, the BDM dynamics supports a truncated power-law distribution [here 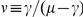],
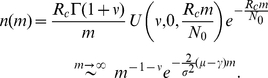
(2)
2. When 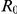 individuals are sampled the effective strength of the sampling is 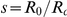. In the strong sampling limit, 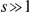, the new distribution is just a rescaled Kummer [15]. On the other hand if 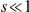,
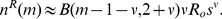
(3)

Eq. (3) implies that the BDM statistics crosses over to the Yule-Simon result when the sampling is weak [see Eq. (1) and the discussion in methods (B)]. Since weak sampling yields mainly members of large families for which the chance of extinction is small, Yule's theory with a net birth rate becomes adequate. Indeed, in the main part of Fig. 1 we show how the BDM Kummer statistics fits the contemporary data for Cerambycinea and how one can reconcile the Yule result by taking into account the effect of sampling. Note that our theory [15] is based on a Fokker-Planck equation that fails when the size of the family is of order unity [22], thus here and in the following figures the curve fails to fit the number of singletons.

**Figure 2:**
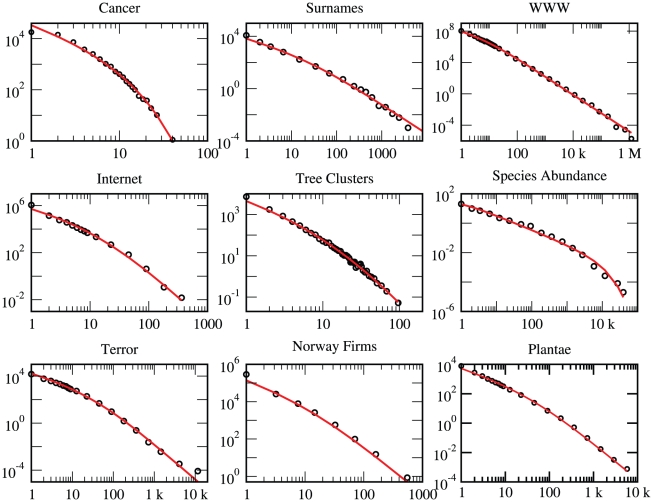
Tour de force of BDM statistics: Pareto plots are presented for empirical datasets obtained from independent studies across many disciplines. The best fit values of 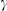 and 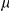 are given for each item. (a) Distribution of number of chromosome abberations in cancer tumors [26]. 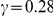 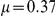 (b) Surname statistics from the 1790 US census. The growth rate (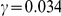) was inferred [15] from historical censuses in England, and the fit retrieves the “mutation” (surname changes) rate to be 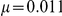. (c) WWW: number of sites with certain degree of links as a function of the degree. The set of 200 million web pages with 1,500 million hyperlinks first considered by Broder et. al. [31] has been analyzed. 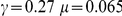. (d) Internet (physical structure) - number of nodes with 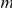 links vs. m. Data obtained from DIMES web site (www.netdimes.org). 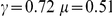. (e) Clusters of trees in the tropical forest. Shown here is the number 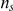 of clusters of size 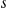 for *Hybanthus pronifolius*, the most frequent species in the Barro-Colorado Island plot [32]. (f) Species abundance ratio in the tropical forest [32]. Here 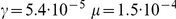. (g) Human insurgency: number of terror attacks with 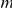 casualties vs. 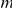. Data from Global Terrorism Database, START (http://www.start.umd.edu). 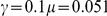. (h) Number of Norwegian firms with 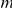 employees, as obtained from statistics Norway website, www.ssb.no. (Data for 2010). 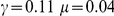. (i) Species within genera statistics for the Plantae kingdom [24] 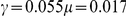.

Fig. 2 demonstrates the power of our technique using many paradigmatic fat-tailed distributions from the social sciences (surnames, insurgency, WWW), engineering (internet), ecology (species within genera, species abundance ratio, clusters of trees), biology (cancer abberations statistics) and economy (firms size distribution). In all cases presented here a two parameter fit is shown, thus we are not using more fitting parameters than a standard power-law fit. In some cases the relevance of the BDM dynamics to the underlying process is clear; in other cases (terror attacks) the underlying process is not well understood, and more studies are needed in order to prove, or disprove, the relevance of BDM, perhaps along the lines suggested by [23]. The agreement of theory and data is impressing with respect to other fits on log-log scale; some examples of other fitting functions and distributions are given in the methods section (D).

Clearly the BDM theory is much stronger than a simple power-law fit, yielding sharper predictions and fitting almost perfectly many paradigmatic empirical datasets. Its amazing success, even where the BDM process is certainly a crude approximation for the real dynamics, suggests that this distribution behaves like a central limit for many multiplicative neutral processes.

For any of the topics of Fig. 2 a comprehensive discussion is needed in order to put our new results for 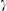 and 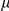 in the context of the specific field. This is beyond the scope of this Letter, and short specific comments are presented in methods, subsection (C).

**Figure 3:**
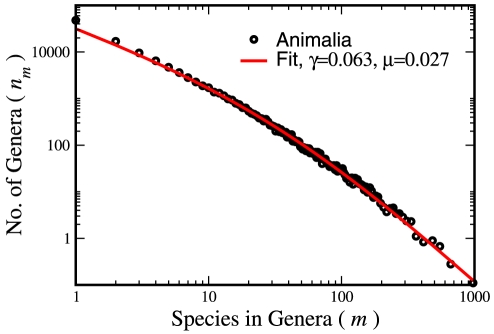
A Pareto plot for the species within genera statistics for the Animalia kingdom. The fit of the BDM theory to the data is surprisingly good, given the existence of different taxonomical classifications for genera. The fit suggests a diversification (speciation minus extinction) rate of about 0.063; this value falls within the confidence intervals obtained by Ricklefs [25] for North and South American clades of passerine birds.

Let us conclude by demonstrating the quality of our results using one example. Figure 3 shows the species within genera statistics for all the Animalia kingdom [24]. The Kummer function fits almost exactly the empirical data, much better than other distributions conjectured (see SM). The rate of diversification (speciation minus extinction), 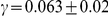, is consistent with the range of values estimated from lineage through time plots [25], and our confidence intervals are much tighter.

## Materials and Methods

### A. The birth-death-mutation process

The birth-death-mutation (BDM) process, in its simplest form, governs the dynamics of 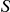 families of agents. Each family is characterized by 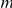, the number of agents in it. For the sake of concreteness let us consider a population of species (agents), each of which belongs to a genus (family).

At every time step a species is chosen at random among all species, independent of its genus. This agent is removed with probability 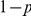 and reproduces (speciates) with probability 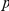. The offspring belongs to the same genus as its parent species with probability 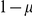, and “mutates” to form a new genus with probability 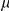. Note that we use the word “mutation” to indicate an offspring that forms a new family (genus, surname), rather than belonging to the same clan as its parent. The parameter 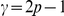 defines the growth rate (if positive) or the decay rate (if negative) of the population. This is the overlapping generations (Moran) version of the process.

Many other processes support the same steady state distribution of family sizes [15]. Of particular importance is the nonoverlapping generations (Wright-Fisher) version of this dynamics. In this case all agents produces offspring at once and then are removed. An agent produce 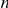 offspring with probability 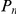. The average number of offspring per individual is thus given by 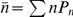, and the growth/decay rate is 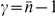. Again 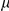 is the mutation rate as described above.

In previous work [15] we have shown that all these processes yield the same steady-state distribution of family sizes, which is independent of the “microscopic” details. The final distribution depends only on the growth rate 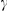, the mutation rate 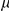, and the variance 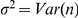. For the Moran case 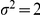. It turns out that 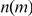 satisfies the Kummer differential equation
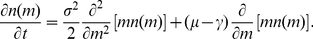
(4)Note that this equation resembles a diffusion-convection process for 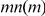.

The same statistics emerges if agents are removed with probability 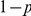, reproduce into the parent set with probability 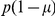, and new agents, each deposited into an empty set (family), are added with probability 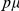 (we refer to this as the birth-death-source process, BDS). This is the case, e.g., if nodes, each carrying a certain number of links, are added to an already existing network and the chance of a link to be attached to an already existing node is proportional to the degree of the node. If links are removed at a different rate, the process yields the same statistics as the BDM (up to slight modifications since new families appear, in realistic networks, with size which is greater than one).

The BDM process is a generalization of the famous Yule process which has no death in it; i.e., agents are only born and mutate. In the same sense, the BDS version generalizes the preferential attachment process [16] in which links are only added to the network but are never removed.

### B. Yule-Simon statistics as a weak sampling limit of BDM

In the process defined by Yule there is no death, and the mutation rate 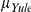 is simply the ratio between the average number of new surnames (or genera) that appear during a period of time and the number of new individuals added, during the same period, to already existing families (see the detailed discussion in [11]).

In the BDM process the rate in which new families are generated is 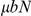 (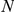 is the total population at certain time, 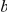 is the birth rate) and the rate in which the total population in the already existing families grows is 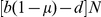. Without loss of generality we can choose 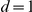 such that 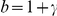, since the growth rate 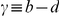. The ratio between the new families generation rate and the old families growth rate is, (to the first order in the small parameters 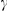 and 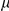), 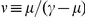. This implies that for small growth and mutation rates, which is the regime of validity of the Kummer theory, Yule theory is equivalent to BDM iff stochastic extinction is neglected and 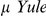 is replaced by 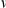. For that reason, Eq. (5) of the main text is equivalent to Yule statistics (Eq. 1) with 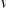 instead of 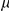.

### C. 3 Remarks for Figure 2 of the main text

The remarks below refer to the panels of Fig. 2:

General: The binning of the data was done using a half logarithmic scale, which means that for small families (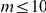) we had a bin for every number, while for large families we used logarithmic binning with a bin size 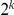 (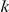 is the bin number). We have found this to be optimal in terms of presentation clarity, but the Kummer fit has been checked using other binning schemes and the differences are negligible. For two datasets (surname panel (b), and firms panel (h)) the data was available only in a binned form, so the existing binning scheme has been retained.

- Cancer: The data we present here is the distribution of the number of chromosome abberations in cancer tumors [26], includes all different types of cancer. See [27] for analysis of different types of cancer.
- Surname: The size of a family was defined as the number of households having the same surname. Data refer to the US census of 1790, when the US population shared the same genealogic and demographic histories with the British population. The English demography is roughly documented since the Domesday Book census carried out by William the Conquerer. For more details see [15].
- WWW links statistics. There is some ambiguity about the kind of sampling involved in the collection of the data. In principle one should make a distinction between building a surname statistics by sampling *individuals* and asking for their surname, in which case Eq. (5) of the method section is applicable, and sampling surnames and asking for the number of individuals having this specific surname. In the internet case the sampling is done by crawlers moving from node to node along the links; here a link is an individual and a node is a “surname”. In any case, the success of our fit to a full census theory means that the effect of sampling, if any, is weak (i.e., that we are in the strong sampling regime).
- We present here the nodes in-degree distribution (i.e. the size of a node is determined by the number of links pointing to it). The nodes out-degree distribution does not follow Kummer. This difference needs further analysis.
- The data presented here is for the most frequent species in the plot, *Hybanthus pronifolius*. There are about 40000 individual trees of this species in a rectangular area of 1000×500 meters. We have covered the area by a 2×2 meters grid and consider any square that contains at least a single *Hybanthus* tree as black, other squares are white. We then identified and tracked black cluster using the standard Hoshen-Kopelman algorithm. We have checked that the results are not sensitive to slight modifications of the lattice constant (grids with 1–3 meters squares were checked) and have gotten fits of similar quality for the other frequent species in the plot.
- The data was averaged over six different censuses. Time between consecutive censuses is five years, to be compared with the lifetime of a tree which is typically about 100 years.
  Our best fit yields 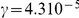 and 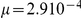. This suggests that the total population of the meta-community isn't really fixed but rather grows extremely slowly. Although the model is neutral, the overall effect of adaptation may very slowly increase the carrying capacity of the forest.
  While we are not trying to claim that our fit is actually conclusive, this result opens an interesting possibility for refutation of the critics of the “point mutation” version of Hubbell's theory, who base themselves on turnover rates. As pointed out by Ricklefs [28] and by Nee [29] the time to origination of a species with N individuals is about 2N. This leads to ridiculously large timescales when applied to realistic species abundance. One implication of our work is that the introduction of a very weak growth rate does not kill the statistics, yet it clearly shortens the time to origination significantly. For example for 10 million trees with generation time of a 100 years, the time to origination if the total population is fixed will be of order of a billion years, while for the 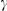 above it will be 40 million years.
- The datasets had also some non-integers values (the meaning of which is unclear to us) that we rounded up to the closest integer number.
- The dataset includes the number of establishments with 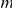 employees, starting from 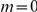. In order to avoid this zero we have shifted 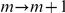, counting the owner also as an employee.
- The statistics of the Plantae kingdom. This dataset is similar to the Animalia displayed and analyzed in Fig. 3; we have preferred to present a more detailed analysis of Animalia since this is the largest kingdom.

### D. The adequacy of Kummer

When dealing with fat-tailed distributions that are extended over many orders of magnitude, a log-log plot must be used. However, these plots are notoriously known to smear out some fine details of the distribution, and sometimes this feature blurs the actual mismatch between the theory and the empirical data. The level of exactness is thus a crucial factor in determining the adequacy of a fit. Here we describe two examples.

**Figure 4:**
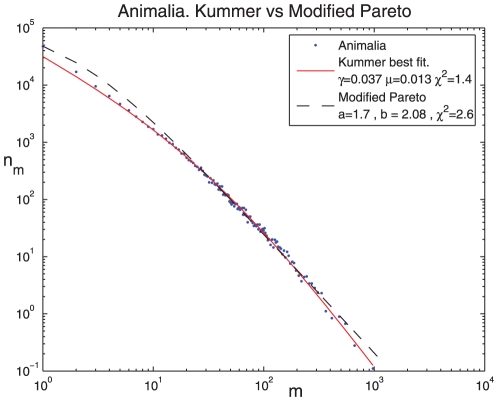
Animalia kingdoms statistics: Modified pareto (Zipf-Mandelbrot, dashed line) best fit vs. Kummer best fit.

First, in Fig. 4 the Kummer best fit is compared with the best fit obtained for the modified Pareto (Zipf - Mandelbrot) distribution, which is a two parameter law with the same concave shape,
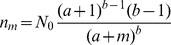
(5)where 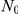 is the population size. The best fit for the parameters 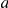 and 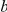 is shown together with the best Kummer fit. One can see that, although the mismatch is never large in a loglog plot once the function captures the general trend, there are systematic deviations in the modified Pareto case but not from the Kummer function (note again that the singletons are not covered by our theory so the mismatch at 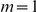 is irrelevant).

**Figure 5:**
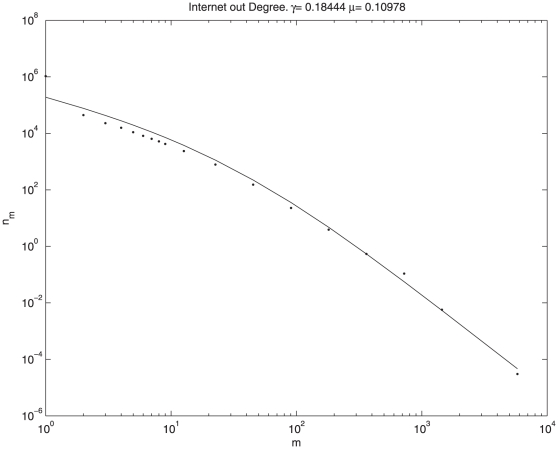
Out-degree statistics: The best fit to Kummer fails systematically at small 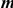
s.

As another example let us present a case where systematic deviations from Kummer show up. In Fig. 5 the out-degree distribution of nodes in the internet (the in-degree that satisfies Kummer is shown in Fig. 2d) is shown together with the best fit to Kummer, and indeed one can see systematic deviations that makes the Kummer fit very suspicious, if not fully disqualified.

In general the Kummer function may be considered in any case where the distribution is monotonically decreasing (so it is inappropriate as an explanation to, say, scientific citation statistics where a hump appears at intermediate values of 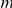). For a reasonable fit the slope at small 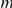-s should be close to one, not too shallow (as in the Tsallis distribution [30]) or too steep.
